# Comparison of caries detection methods using varying numbers of intra-oral digital photographs with visual examination for epidemiology in children

**DOI:** 10.1186/1472-6831-13-6

**Published:** 2013-01-11

**Authors:** Uriana Boye, Ian A Pretty, Martin Tickle, Tanya Walsh

**Affiliations:** 1 The Oral Health Unit, School of Dentistry, University of Manchester, Manchester Academic Health Sciences Centre, Manchester, UK; 2 Dental Health Unit, 3A Skelton House ,Lloyd Street North, Manchester, UK; 3 The Cochrane Oral Health Group, University of Manchester, Manchester Academic Health Sciences Centre, Manchester, UK

**Keywords:** Intra-oral photographs, Caries, Visual examination, Dental epidemiology

## Abstract

**Background:**

This was a method comparison study. The aim of study was to compare caries information obtained from a full mouth visual examination using the method developed by the British Association for the Study of Community Dentistry (BASCD) for epidemiological surveys with caries data obtained from eight, six and four intra-oral digital photographs of index teeth in two groups of children aged 5 years and 10/11 years.

**Methods:**

Five trained and calibrated examiners visually examined the whole mouth of 240 5-year-olds and 250 10-/11-year-olds using the BASCD method. The children also had intra-oral digital photographs taken of index teeth. The same 5 examiners assessed the intra-oral digital photographs (in groups of 8, 6 and 4 intra-oral photographs) for caries using the BASCD criteria; dmft/DMFT were used to compute Weighted Kappa Statistic as a measure of intra-examiner reliability and intra-class correlation coefficients as a measure of inter-examiner reliability for each method. A method comparison analysis was performed to determine the 95% limits of agreement for all five examiners, comparing the visual examination method with the photographic assessment method using 8, 6 and 4 intra-oral photographs.

**Results:**

The intra-rater reliability for the visual examinations ranged from 0.81 to 0.94 in the 5-year-olds and 0.90 to 0.97 in the 10-/11-year-olds. Those for the photographic assessments in the 5-year-olds were for 8 intra-oral photographs, 0.86 to 0.94, for 6 intra-oral photographs, 0.85 to 0.98 and for 4 intra-oral photographs, 0.80 to 0.96; for the 10-/11-year-olds were for 8 intra-oral photographs 0.84 to 1.00, for 6 intra-oral photographs 0.82 to 1.00 and for 4 intra-oral photographs 0.72 to 0.98. The 95% limits of agreement were −1.997 to 1.967, -2.375 to 2.735 and −2.250 to 2.921 respectively for the 5-year-olds and −2.614 to 2.027, -2.179 to 3.887 and −2.594 to 2.163 respectively for the 10-/11-year-olds.

**Conclusions:**

The photographic assessment method, particularly assessment of 8 intra-oral digital photographs is comparable to the visual examination method in the primary dentition. With the additional benefits of archiving, remote scoring, allowing multiple scorers to score images and enabling longitudinal analysis, the photographic assessment method may be used as an alternative caries detection method in the primary dentition in situations where the visual examination method may not be applicable such as when examiner blinding is required and in practice based randomised controlled trials (RCTs).

## Background

Although there has been an improvement in oral health, levels of dental caries remain high in some sections of society and caries is still the most significant cause of poor oral health in children [1]. Dental caries epidemiological surveys, as well as studies designed to evaluate the effectiveness of interventions for caries prevention and management are therefore mainly, although not exclusively, conducted in children. Having the appropriate tools to support the delivery of reliable dental epidemiological surveys and enable robustly designed studies to be conducted is therefore important.

In the UK the National Health Service (NHS) dental epidemiological surveys which are regularly undertaken have ensured that the UK has one of the most respected caries surveillance programmes for children. These UK surveys use the well documented visual examination method developed by the British Association for the Study of Community Dentistry (BASCD) [2]. However visual dental examinations by their nature can introduce assessment bias into dental epidemiological studies and therefore limit their robustness. This is particularly relevant when examiner blinding is required in studies undertaken to evaluate of oral health intervention strategies or community water fluoridation schemes [3]. Having considered the barriers to using other methods of caries detection as an alternative to the visual examination method, a study by Boye et al. [4] showed that assessments of intra-oral photographs has promise. Intra-oral photographs have been used in the clinical setting to record caries and hypo-mineralization in primary molars [5] and to score caries on primary and permanent teeth in the epidemiological setting [6]. The use of intra-oral cameras in the epidemiological setting has been shown to be acceptable to children, the main population involved in caries epidemiological and intervention studies [7]. An advantage that the use of intra-oral photographs has over visual examination methods in such studies is the ability to archive intra-oral photographs. This permits multiple scorers to score the images as well as remote scoring and longitudinal analysis.

There are however surmountable practical challenges to more widespread use of this method of data capture. Examiners who trialled this photographic assessment method were found to be optimistic about the use of this method in dental epidemiology with improved utility [6]. One of the key challenges relates to the number of intra-oral photographs required to provide adequate information to undertake a caries assessment. To enable the assessment of all surfaces of the teeth as they would be in a visual examination of the mouth, examiners have to be provided with intra-photographs showing all surfaces of all the teeth. This approach makes the photographic assessment method a much longer and therefore more costly process as compared to visual examination methods.

The evidence from the UK NHS dental epidemiological surveys data 2002 – 2010 (The Dental Observatory, Preston UK) show that caries in the primary dentition is usually located in the molars, upper incisors and lower canines and caries in the permanent dentition is usually found in the first molars and so it would make sense pragmatically and financially to limit the data capture to these teeth assuming there is no loss of information required to support assessment. These teeth are henceforth referred to as the *index* teeth. Use of index teeth or sites is common in other epidemiological and clinical assessments, for example in caries and periodontal studies [8,9].

The aim of the study was to compare caries information obtained from a full mouth visual examination using the method developed by the British Association for the Study of Community Dentistry (BASCD) for epidemiological surveys with caries information obtained from eight, six and four intra-oral digital photographs of index teeth in two groups of children aged 5 years and 10-/11 years.

The Objectives were to test:

the intra-examiner reliability of all examiners for each method

the inter examiner reliability for each method, and

the agreement between the examination methods by comparing the mean caries indices obtained using the visual method with

○ the mean caries indices obtained from the assessment of eight intra-oral photographs of identified teeth liable to decay (index teeth) in the same subjects

○ the mean caries indices obtained from the assessment of six intra-oral photographs of index teeth in the same subjects

○ the mean caries indices obtained from the assessment of four intra-oral photographs of index teeth in the same subjects

in two groups of children aged 5 years and 10/11 years; the two main cohorts examined in the UK NHS epidemiological surveys.

## Methods

Ethical approval was obtained for the study from the National Research Ethics Service, UK (Reference Number: North West 10 09/H1011/57).

This was a cross-sectional, method comparison study comparing an established visual examination method developed by the British Association for the Study of Community Dentistry (BASCD) for the nationally coordinated NHS epidemiological surveys of children in the UK with a photographic assessment method in a sample of 5-year-old and 10-/11-year-old children.

### Study population

Five-year-old and 10-/11-year-old children attending state primary schools in Rochdale an area in the North West of England, with a 5-year-olds population dmft of 2.08. dt of 1.79, mt of 0.17 and ft of 0.12 in 2007/2008 and 12-year-old population DMFT of 0.95, DT of 0.40, MT of 0.09 and FT of 0.45 (The Dental Observatory, Preston UK), was the study population. Before data was collected, study invitation letters, study information sheets and consent forms were sent to parents/legal guardians of eligible children via their children’s schools, informing them about the study. Parents/guardians were asked to provide informed consent to enable their child to participate. Completed consent forms were returned to the study team via the schools. For the 5-year-olds, only children whose parents or legal guardians gave positive consent were included in the study. For the 10-/11-year-olds in accordance with the guidance from the UK Department of Health [10] regarding consent for this age group, only those children who gave informed consent in addition to their parent’s compliance to let them participate were included in the study. Each child recruited into the study was assigned a unique study identification number (ID).

### Examination and assessment

The children in each age group had a visual dental examination according to BASCD diagnostic protocol [11] and also had 8 intra-oral photographs taken of their dentition on the same day as the visual examinations. All the examiners involved in the study were experienced epidemiological examiners (with a minimum of 10 years’ experience) and had been trained and calibrated to the BASCD caries examination protocol as members of the UK National Epidemiological Surveys team. [12] Completion of this national training and calibration based on a minimum sensitivity of 0.75 a specificity of 0.90 for the primary teeth and a minimum sensitivity of 0.80 a specificity of 0.90 for the permanent teeth was used as the main selection criterion for the examiners used in this study.

### Visual dental examinations

The visual examinations were performed by 5 dentists one of whom was a bench mark examiner for the UK NHS epidemiology programme. All the dental examinations took place in the children’s schools. During the visit to each school five examination stations were set up to enable 5 children to be examined at a time in each examination cycle. The children lay supine on each of five examination tables with an examiner seated at the head end. The children remained at the examination stations whilst the examiners moved round the stations, examining each child in turn until all the children had been assessed by all 5 examiners. At the end of each examination cycle another group of 5 children were brought to the stations to replace those already examined for a new examination cycle to begin. The examination for dental caries was carried out according to the method, criteria and coding system employed in the BASCD coordinated NHS Epidemiology Programme [11], using the recommended instrumentation and equipment: Daray X100 Lamps with Pivot D desk mount (Daray Healthcare Products® Swadlincote, Derbyshire) as light source, a hand mirror, cotton wool rolls and a blunt probe for the removal of debris and the sterilisation/disinfection precautions. Data collection and data validating methods as stipulated by BASCD [13] were applied using Dental Survey Plus (Dental Survey Plus 2® The Dental Health Services Research Unit, University of Dundee).

The primary teeth were examined in the 5-year-olds and only the erupted permanent teeth were examined in the 10-/11-year-olds. All surfaces of each eligible tooth examined were scored. Caries was diagnosed visually at the ‘caries into dentine’ level. 15% of the children in each age group were re-examined to test intra-examiner reliability. The scores for each subject were recorded by a scribe onto a pro-forma labelled with the unique ID of that subject and inputted into Dental Survey Plus 2® software programme.

### Photographic procedures and assessments

Prior to taking the intra-oral photographs, electronic folders carrying the same unique IDs as those assigned to the subjects for the visual examinations were created on a password protected computer. This was to enable matching of the visual examination and photographic assessment scores during analysis.

An intra-oral camera, the Sopro 717 (The Acteon Group® Eaton Socon, Cambridgeshire), with its own integral light-emitting diode (LED) light source, was used to take 8 intra-oral digital photographs of the index teeth for each subject. The index teeth for the 5-year-olds were all first and second primary molars, the upper central and lateral primary incisors and the lower primary canines. The index teeth for the 10-/11-year-olds were all four first permanent molars (intra-oral photographs were obtained showing the occlusal and buccal surfaces of the lower first permanent molars; occlusal and palatal surfaces of the upper first permanent molars).

**Figure 1:**
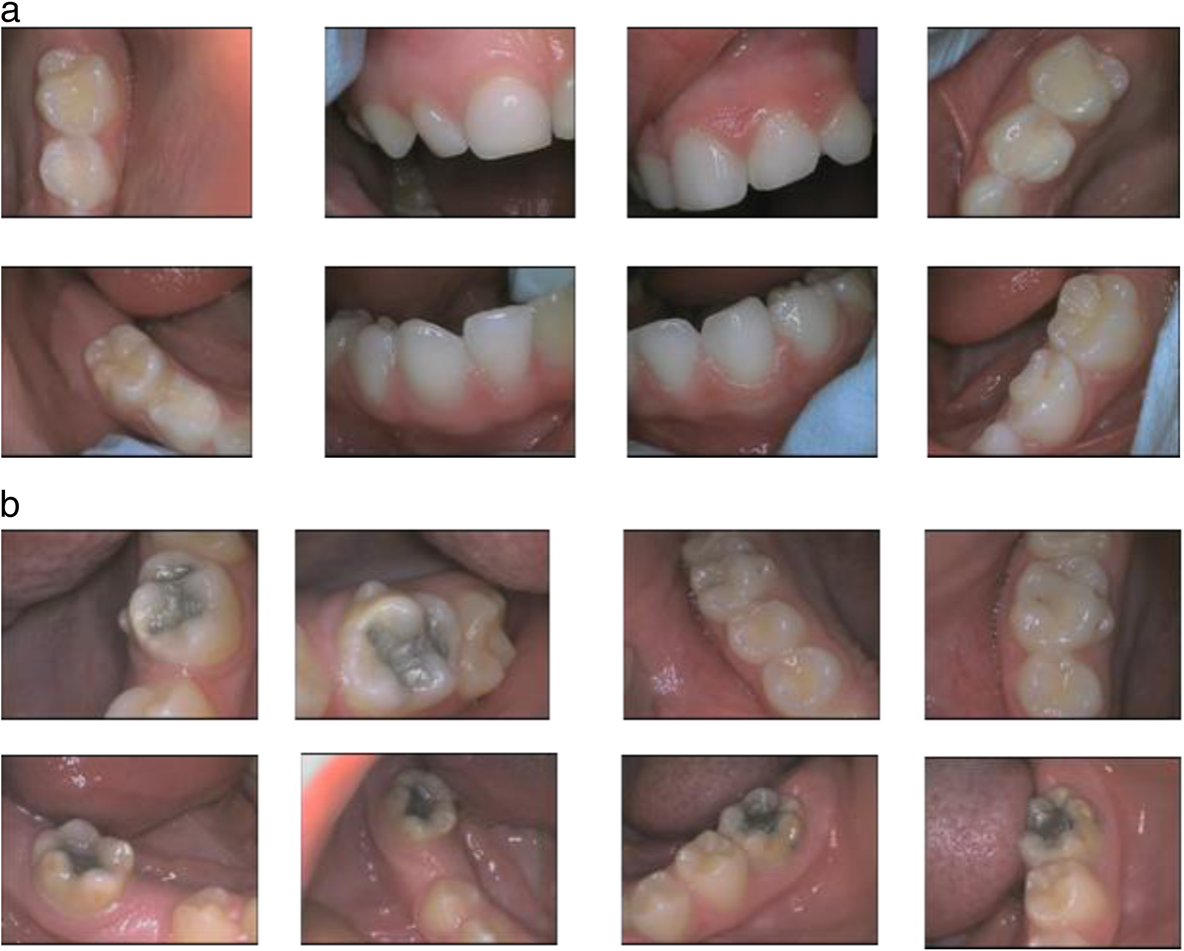
a Example of 8 intra-oral photographs of a 5-yr-old. b Example of 8 intra-oral photographs of a 10/11-yr-old.

The intra-oral camera system was connected to a laptop computer with bespoke software package. This system allowed each captured intra-oral digital photograph to be previewed before saving it to specified tooth labelled slots in the subject’s allocated electronic folder. The intra-oral photographs (Figures 1a and 1b) were obtained on the same day as the visual examinations. Each child lay supine on an examination table with the examiner/photographer seated behind them at the head end. The teeth were dried with cotton wool rolls (following the same procedure as the visual examinations) prior to taking the intra-oral photographs. To obtain the photographs, the camera was held by the operator in one hand using a dental hand-piece grip with the LED tip of camera pointing towards the tooth surfaces to be photographed. The fingers of the other hand were used to support the subjects’ jaws as well as when required for the retraction of the checks and tongue to allow access to the teeth surfaces. The tip of the camera was moved relative to the position of the tooth to ensure that the required tooth surface to be photographed stayed in focus at the same zoom. The use of a foot control allowed the generated digital images to be saved or discarded. The “intra-oral” selection setting of the camera was used. The photographs were saved as Bitmap images. Between subjects, the infection control procedures specified by the manufacturer of the intra-oral camera’s user guide was followed.

Using the labelled electronic folders containing the 8 intra-oral photographs for each subject, two new electronic folders were created containing 6 and 4 intra-oral photographs respectively by selectively removing photographs in a standardized way. Two intra-oral photographs of the upper central and lateral incisors were removed and the resulting folder with 6 intra-oral photographs was renamed with the ID label of the subject but with the suffix 6 added. The same process was used to create the electronic folders with 4 intra-oral photographs. Starting with the electronic folder containing the 6 intra-oral photographs, the two intra-oral photographs of the lower left and lower right primary canines were removed leaving 4 intra-oral photographs showing all the primary molars. For the permanent teeth, two intra-oral photographs showing the palatal surfaces of the upper first permanent molars were removed for each child to produce the folders with 6 intra-oral photographs. Then a further two intra-oral photographs showing buccal surfaces of the lower first permanent molars were removed for each child to produce the folders with 4 intra-oral photographs. When the compilation of the folders was completed, there were three folders for each subject: ID labelled (8), ID labelled (6) and ID labelled (4) containing 8, 6 and 4 intra-oral photographs respectively.

In total 6 photographic electronic folders (5-year-olds: 8 intra-oral photographs per subject, 5-year-olds: 6 intra-oral photographs per subject and 5-year-olds: 4 intra-oral photographs per subject; 10-/11-year olds: 8 intra-oral photographs per subject, 10-/11-year-olds: 6 intra-oral photographs per subject and 10-/11-year-olds: 4 intra-oral photographs per subject) were prepared for assessment and loaded onto USB flash drives. For each of the presentations, 15% of the ID labelled folders were assigned new ID numbers and added to the presentations. This was to test intra-examiner reliability of the photographic assessments. The key to the original identity numbers and the new identity numbers (for those added to test intra-examiner reliability) were retained by the study administrator. The intra-oral photographs were not printed out; they were viewed and assessed as digital photographs.

The same 5 examiners who had examined the children visually assessed the intra-oral digital photographic presentations of the children’s dentitions, blinded to the results of their visual assessment. Before undertaking the photographic assessments all the examiners convened to receive training on the process of viewing the intra-oral photographs and navigating through the electronic folders using windows explorer but were not calibrated in assessing the intra-oral photographs. Each examiner was provided with a USB flash drive with the electronic folders with the photographs four weeks after the visual examinations. Each examiner viewed the intra-oral photographs on computer screens at a time of day and room conditions of their choice but all of the examiners used windows explorer for viewing the digital photographs. As was the case for the visual examination, caries was diagnosed using the BASCD diagnostic criteria. The examiners recorded the scores from their intra-oral photographic assessments for each subject onto a paper pro-forma, identical to the one used for the visual examination.

### Data processing and analysis

The data collated from the visual examinations and intra-oral photographic assessments of the subjects’ teeth were entered into Dental Survey Plus 2® (DSP2) software programme (The Dental Health Services Research Unit, University of Dundee). The software was used to analyse the data and generate mean caries experience indices at tooth level i.e. dmft and components (dt, mt, ft) and DMFT and components (DT, MT, FT) for the deciduous and permanent dentition respectively. The Weighted Kappa statistic was used as a measure of intra-rater reliability for both the visual examinations and the photographic assessments in both age groups and the Landis and Koch measurement of observer agreement for categorical data [Landis and Koch, 1977b] was used to determine the level of agreement.

The mean caries indices data generated by DSP2 software was exported into Stata® statistical software version 11 (Stata Corporation, Texas) to compute intra-class correlation coefficients as a measure of inter-examiner reliability for each method. A method comparison analysis was performed using Stata® version 11 to determine the 95% limits of agreement for all five examiners, comparing the visual examination method with the photographic assessment method using 8, 6 and 4 intra-oral photographs [14].

Using the difference in mean dmft/DMFT values as a measure to determine the bias between the methods, a priori estimate of mean dmft/DMFT value within ± 0.3 was set as an acceptable difference for the samples overall.

## Results

A total of 240 5-year-olds and 250 10-/11-year-olds were recruited into the study. Of these, 39 5-year-olds and 19 10-/11-year-olds did not have both visual examination and intra-oral photographs taken of their dentition. Their data were therefore excluded from analysis.

The weighted kappa statistic computed as a measure of intra-rater reliability showed almost perfect agreement for all the examiners using the different examination and assessment methods. The weighted kappa statistic for the visual examinations ranged from 0.81 to 0.94 with a median value of 0.93 in the 5-year-olds and 0.90 to 0.97 with a median value of 0.92 in the 10-/11-year-olds. The weighted kappa statistic for the photographic assessments in the 5-year-olds were for 8 intra-oral photographs, 0.86 to 0.94 (median 0.94), 6 intra-oral photographs, 0.85 to 0.98 (median 0.94) and for 4 intra-oral photographs, 0.80 to 0.96 (median 0.93). The weighted kappa statistic for the photographic assessments in the 10-/11-year-olds were for 8 intra-oral photographs 0.84 to 1.00 (median 0.91), 6 intra-oral photographs 0.82 to 1.00 (median 0.91) and for 4 intra-oral photographs 0.72 to 0.98 (median 0.92).

Table 1 shows the computed intra-class correlation coefficients (ICC) as a measure of inter-rater reliability of the methods. The agreement within the group of examiners for the 5-year-olds’ assessments was high for all methods. In the 10-/11-year-olds the ICC was higher within the group of examiners for the visual examination method as compared to the intra-oral photographic assessment methods.

**Table 1:** Intra-class correlation coefficient as a measure of inter -examiner reliability

	**Intra-class correlation coefficient (inter-examiner reliability) with 95% confidence interval**			
**Population**	**Visual**	**8 Photos**	**6 Photos**	**4 Photos**
**5-year-olds dmft**	0.963 (0.954 to 0.970)	0.964 (0.956 to 0.971)	0.870 (0.817 to 0.906)	0.958 (0.948 to 0.967)
**10/11-year-olds DMFT**	0.896 (0.875 to 0.914)	0.584 (0.525 to 0.642)	0.764 (0.710 to 0.809)	0.531 (0.470 to 0.592)

Tables 2 and 3 show the summary of the mean indices with standard deviations computed from the scores of the individual examiners for all the examination and assessment methods for the 5-year-olds and the 10-/11-year-olds respectively.

**Table 2:** The mean caries indices with standard deviations according to examination method in 5-year-olds

**Examiner**	**Mean dt**				**Mean mt**				**Mean ft**				**Mean dmft**			
	**V**	**8P**	**6P**	**4P**	**V**	**8P**	**6P**	**4P**	**V**	**8P**	**6P**	**4P**	**V**	**8P**	**6P**	**4P**
Bench Mark	1.69 ± 2.69	1.75 ± 2.67	1.72 ± 2.64	1.33 ± 2.15	0.33 ± 1.54	0.33 ± 1.54	0.33 ± 1.54	0.33 ± 1.50	0.08 ± 0.38	0.08 ± 0.42	0.08 ± 0.47	0.07 ± 0.46	2.11 ± 3.07	2.14 ± 3.04	2.13 ± 3.05	1.70 ± 2.15
1	1.85 ± 2.68	1.69 ± 2.56	1.69 ± 2.65	1.36 ± 2.15	0.33 ± 1.54	0.33 ± 1.54	0.33 ± 1.54	0.33 ± 1.50	0.13 ± 0.50	0.16 ± 0.55	0.15 ± 0.58	0.15 ± 0.54	2.31 ± 3.12	2.15 ± 3.03	2.17 ± 3.08	1.85 ± 2.15
2	1.92 ± 2.90	2.00 ± 2.87	2.12 ± 2.92	1.69 ± 2.37	0.33 ± 1.54	0.33 ± 1.54	0.33 ± 1.54	0.33 ± 1.51	0.11 ± 0.49	0.08 ± 0.42	0.06 ± 0.37	0.07 ± 0.31	2.35 ± 3.27	2.42 ± 3.22	2.53 ± 3.26	2.06 ± 2.37
3	1.70 ± 2.69	1.77 ± 2.66	1.71 ± 2.64	1.37 ± 2.19	0.33 ± 1.54	0.33 ± 1.54	0.33 ± 1.54	0.33 ± 1.54	0.11 ± 0.44	0.08 ± 0.42	0.08 ± 0.40	0.09 ± 0.41	2.15 ± 3.10	2.18 ± 3.05	2.12 ± 3.04	1.80 ± 2.19
4	1.84 ± 2.82	1.90 ± 2.73	1.81 ± 2.70	1.54 ± 2.23	0.33 ± 1.54	0.33 ± 1.54	0.33 ± 1.54	0.33 ± 1.54	0.07 ± 0.33	0.07 ± 0.39	0.10 ± 0.44	0.16 ± 0.48	2.25 ± 3.18	2.28 ± 3.10	2.24 ± 3.07	2.02 ± 2.23

V = Visual Examinations P = Photographic Assessments.

**Table 3:** The mean caries indices with standard deviations according to examination method in 10-/11-year-olds

**Examiner**	**Mean DT**				**Mean MT**				**Mean FT**				**Mean DMFT**			
	**V**	**8P**	**6P**	**4P**	**V**	**8P**	**6P**	**4P**	**V**	**8P**	**6P**	**4P**	**V**	**8P**	**6P**	**4P**
Bench Mark	0.50 ± 1.00	0.69 ± 1.07	0.66 ± 1.05	0.61 ± 1.01	0.12 ± 0.58	0.10 ± 0.54	0.10 ± 0.54	0.10 ± 0.53	0.24 ± 0.68	0.20 ± 0.60	0.22 ± 0.62	0.21 ± 0.61	0.87 ± 1.30	0.99 ± 1.30	0.98 ± 1.28	0.92 ± 1.27
1	0.47 ± 0.93	0.50 ± 0.94	0.34 ± 0.81	0.43 ± 0.87	0.12 ± 0.60	0.11 ± 0.57	0.10 ± 0.57	0.10 ± 0.53	0.26 ± 0.66	0.26 ± 0.68	0.27 ± 0.70	0.26 ± 0.68	0.85 ± 1.26	0.88 ± 1.23	0.71 ± 1.16	0.79 ± 1.19
2	0.40 ± 0.85	1.19 ± 1.39	1.08 ± 1.28	0.97 ± 1.24	0.12 ± 0.58	0.10 ± 0.55	0.09 ± 0.55	0.09 ± 0.55	0.23 ± 0.58	0.13 ± 0.48	0.15 ± 0.51	0.16 ± 0.55	0.74 ± 1.19	1.42 ± 1.50	1.32 ± 1.43	1.21 ± 1.38
3	0.48 ± 0.92	0.94 ± 1.22	0.89 ± 1.18	0.82 ± 1.18	0.12 ± 0.58	0.09 ± 0.55	0.09 ± 0.55	0.09 ± 0.55	0.23 ± 0.58	0.19 ± 0.58	0.19 ± 0.60	0.19 ± 0.60	0.82 ± 1.25	1.22 ± 1.39	1.18 ± 1.35	1.11 ± 1.35
4	0.55 ± 1.11	0.87 ± 1.23	0.89 ± 1.31	1.00 ± 1.37	0.12 ± 0.58	0.10 ± 0.55	0.10 ± 0.55	0.10 ± 0.55	0.25 ± 0.58	0.19 ± 0.59	0.20 ± 0.60	0.16 ± 0.54	0.92 ± 1.39	1.17 ± 1.41	1.19±1.46	1.25±1.52

V = Visual Examinations P = Photographic Assessments.

**Figure 2:**
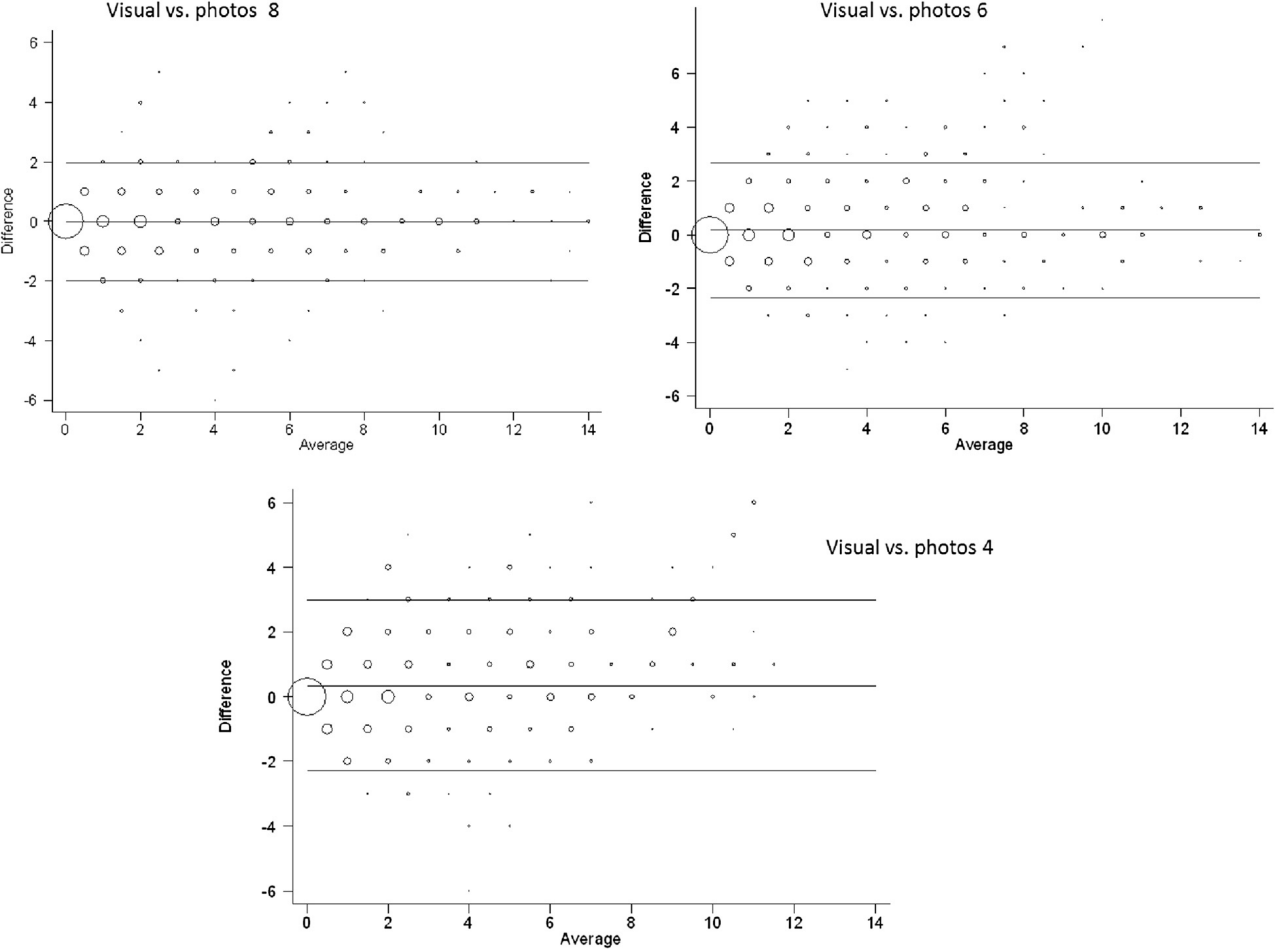
Bland-Altman plots showing limits of agreements for the visual examination and the intra-oral photographic methods in the 5-year-olds.

**Figure 3:**
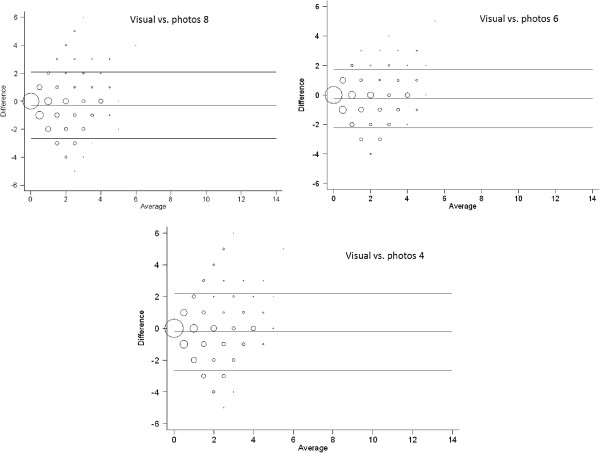
Bland-Altman plots showing limits of agreements for the visual examination and the intra-oral photographic methods for the 10-/11-year-olds.

The 95% limits of agreement comparing the visual examination method with the photographic assessment method using 8, 6 and 4 intra-oral photographs were −1.997 to 1.967, -2.375 to 2.735 and −2.250 to 2.921 respectively for the 5-year-olds and −2.614 to 2.027, -2.179 to 3.887 and −2.594 to 2.163 respectively for the 10-/11-year-olds. Bland-Altman plots were also generated to aid visualization of the limits of agreement between the methods. The corresponding Bland-Altman plots showing the limits of agreement between the methods are shown in Figure 2 for the 5-year-olds and in Figure 3 and the 10-/11-year-olds. Increasing size of the circles on the Bland-Altman plots denotes increasing concentration of observations at the particular points on the plots.

## Discussion

The main findings of the study are that there was very good intra- and inter-examiner reliability for all examination and assessment types in the 5-year-old children with the intra-class correlation coefficient, a measure of inter-examiner reliability for the visual examination method (0.963) similar to that of the assessment of 8 intra-oral photographs (0.964). There was however weaker agreement within the group of examiners when using the photographic assessment method in the 10-/11-year-olds. The narrowest limits of agreement for the 5 year-olds was found between the visual examination and the assessment of 8 intra-oral photographs.

A limitation of this study is that although the examiners were trained and calibrated in the visual examination method, and they received training on the process of viewing the intra-oral photographs, they were not calibrated in assessing the intra-oral photographs. The use of intra-oral photographs however forms part of the standard BASCD training for the visual examination method. Another limitation of the study is that the assessments of the intra-oral photographs were carried out under non-standardised viewing conditions. An in-vitro study that compared assessment of intra-oral photographs under standardised and non-standardised viewing conditions however found no significant difference in outcomes between the two methods [4]. To enable the use of the photographic assessment method in the field, it is necessary for the method to lend itself to pragmatism without detrimental effects on its reliability and validity. Despite the customised viewing conditions, the results of this study show good intra- and inter-examiner reliability for the photographic assessment method especially in the 5-year-olds.

In addition to measures of reliability, other studies in the literature that have compared utility of different caries detection methods have tended to use sensitivity and specificity values as the measures for the comparison [15-17]. Sensitivity and specificity for the photographic method as compared to the visual examination method have been reported [4] to be comparable to or higher than the findings of other caries detection studies [18,19] although there was variation in the stages of caries progression assessed by the different studies. Sensitivity and specificity values as the measures for the comparison of caries detection methods is acceptable when caries prevalence is the only aspect of caries experience that is to be determined. When caries severity is also to be determined as part of such comparisons other measures rather than sensitivity and specificity values may have to be considered for the comparisons.

**Figure 4:**
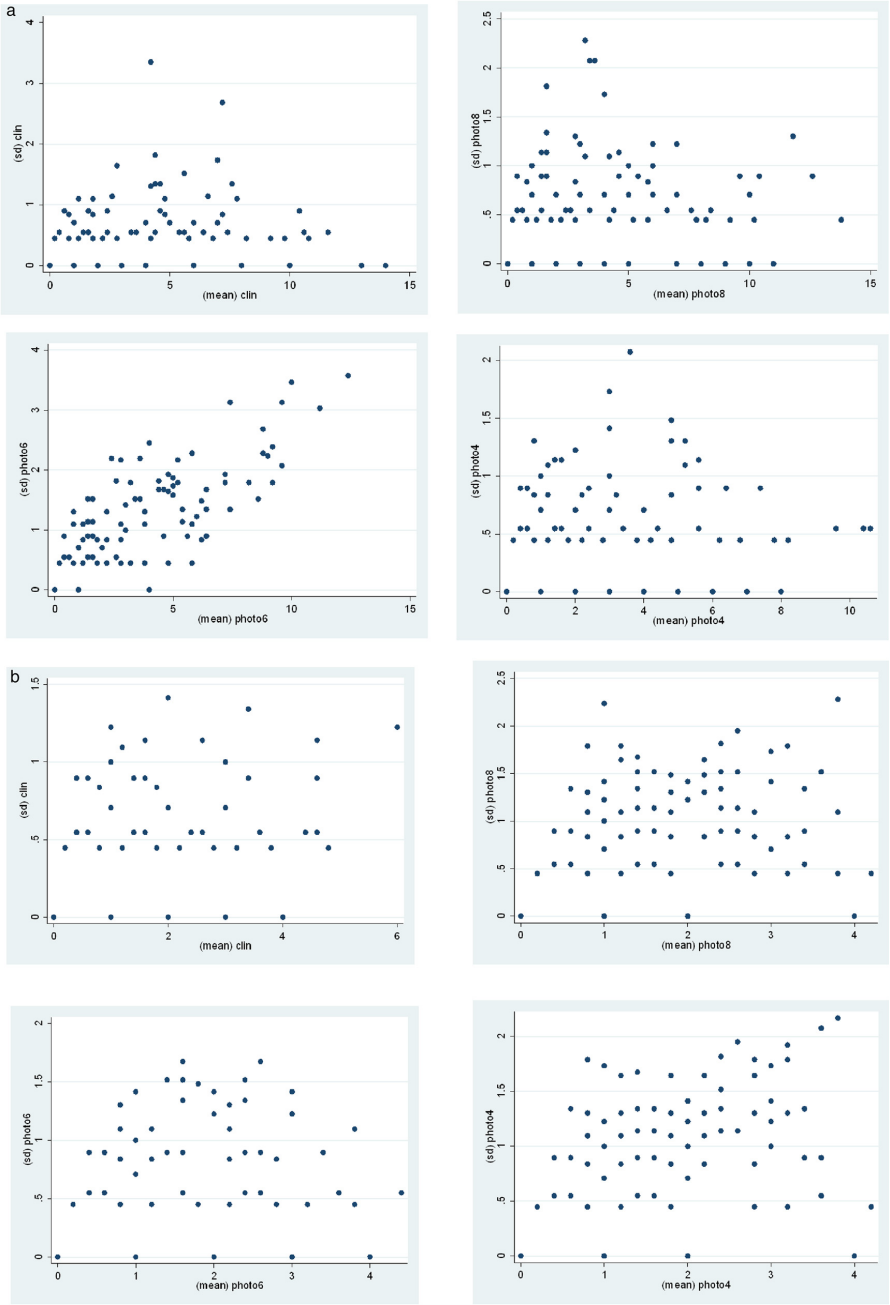
**a Scatter plot of the standard deviations against the means for all methods in all the 201 5-year-olds.b: Scatter plot of the standard deviations against the means for all methods in all the 231 10-/11-year-olds.**

The determination of limits of agreement is used widely in the medical literature to make comparisons between methods of quantifying entities [20]. The limits of agreement between the methods found in the study were wider than the priori estimate of mean dmft/DMFT value to be within ± 0.3 for the samples overall. Solely based on the priori estimate of mean dmft/DMFT value to be within ± 0.3 for the samples overall as the acceptable difference between the visual and photographic methods by this study, the visual examination and photographic assessment method may not be used interchangeably. A detailed examination of the standard deviations from which the limits of agreement were computed however shows that both the visual examination and photographic assessment method showed comparable wide variations in their mean caries indices with relatively large standard deviations (Figures 4a and 4b). This shows that the established visual method was variable in its caries detection ability in this study and the photographic method was no worse than the visual examination method.

The size of the circles on the Bland-Altman plots depicts the number of observations that lie on that point on the plot. The closeness of the largest circles to zero indicates that majority of the DMFT/dmft scores for the individual observations were within the a priori limits. For all pairwise method comparisons however there were many observations which would fall out-with these limits. One possible explanation for this is transcription errors. For example entering the code for “extraction as a result of caries: code 6” instead of the code for “caries-free: code 0” for the molars in all four quadrants of the mouth because of illegible writing would significantly alter the resultant dmft/DMFT value. An electronically integrated formatted pro-forma which allows direct entry of assessment scores coupled with the double entry of data to allow the checking of disagreements, would minimize such transcription errors.

When limits of agreement have been determined is important to decide whether the difference found between the methods being compared is small enough for the particular purpose for which it is intended in practice [21]. As dmft/DMFT is scored per child as whole values it may be tolerable to accept a difference of ± 1 dmft/DMFT for each individual observation. This would however depend on whether the caries detection method is being used to collect data for needs assessments, clinical trial outcomes or disease surveillance. The low systematic errors indicated by the majority of the mean differences approaching zero may make the methods suitable for needs assessments. The magnitude of differences found in this study for some of the individual observations would however make it an unacceptable outcome measure for determining the need for individual dental attention.

The advantages of the photographic assessment method such as archiving, allowing the use of dental skill mix and its use to support training and calibration in dental epidemiology have been well rehearsed. Although solely based on the limits of agreement found in this study the two methods cannot be used interchangeably, the comparability of the population summary measures: the computed mean caries indices for the visual examination and photographic assessment method as well as the consistent high levels of both intra- and inter-reliability of the photographic assessment particularly the assessment of the 8 intra-oral photographs in the deciduous dentition is promising and merits further refinement [6] to promote it use as a potential alternative but not a replacement caries detection method for use in the primary dentition in situations where the visual examination method may not be applicable such as when examiner blinding is required and in practice based RCTs. This should be done with a clear awareness of the differences between the visual examination method and the photographic assessment method.

The visual examination method developed by BASCD against which the intra-oral photographic assessment method has been evaluated in this study is one of many. Of these other caries detection methods, the International Caries Detection and Assessment System index (ICDAS) [22] which takes into account early carious lesions is becoming commonly used and has been for caries epidemiology [23]. The evaluation of the intra-oral photographic assessment method’s ability to detect various levels of caries against these other caries detection methods for example ICDAS system could be tested in further research.

## Conclusion

The photographic assessment method, particularly assessment of 8 intra-oral photographs, was shown to have consistently high levels of intra- and inter-reliability comparable to the visual examination method in the primary dentition. With the additional benefits of archiving, remote scoring, allowing multiple scorers to score images and enabling longitudinal analysis, this method may be used as an alternative caries detection method in the primary dentition in situations where the visual examination method may not be applicable such as when examiner blinding is required and in practice based RCTs. This should be done with a clear awareness of the differences between the two methods.

## Competing interests

None of the authors are aware of any competing interests in the production of this manuscript.

## Authors’ contributions

UB contributed to the protocol, undertook the management of the study, took the photographs and wrote the manuscript. IAP contributed to the protocol, undertook study monitoring and contributed to the manuscript. MT contributed to the protocol, undertook study monitoring and contributed to the manuscript. TW gave statistical advice, assisted with the data analysis and contributed to the manuscript. All authors read and approved the final manuscript.

## Pre-publication history

The pre-publication history for this paper can be accessed here:

http://www.biomedcentral.com/1472-6831/13/6/prepub
